# Obstetrics

**Published:** 1876-05

**Authors:** 


### III. Obstetrics.

1. *Fœtal Death before the Termination of Pregnancy, from a Knot in the Umbilical Cord.* Canivet. (*Le Progrès Méd.*, No. 6.)

A woman in the 8th month of her fifth pregnancy could not walk by reason of extensive abdominal enlargement. Delivery occurred at term of a first fœtus (female) dead, and presenting by the summit, with spontaneous rupture of the bag of waters. Soon after a new pouch of waters presented, ruptured, and was followed by the expulsion of a normal living child.

The first infant was so macerated that the epidermis was readily detached from the dorsum. The belly was retracted, but there was no appreciable lesion ; and in height and volume it corresponded to the living child.

At ten centimeters from the umbilicus a knot was forcibly tied in the cord which was 95 centimeters in length, and wound three times about the neck of the fœtus. The portion of the cord between the neck and the umbilicus was red and congested, while that extending from the knot to the placenta was, in color and appearance, quite normal. The knot itself was closely tied and exhibited a distinct coagulation of blood in the umbilical vessels at that point. The cord was slender, but the placenta normal.

The interest in this case lay in the death of the fœtus, which, judging from the maceration, had occurred several days before birth. The majority of authors have decided that those knots are never sufficiently constricted to interfere with the circulation and induce the death of the fœtus. (Mauriceau, Beaudelocque, Cazeaux, Tarnier.) One single case, (reported by Voetz) has been published in the *Gazette des Hôpitaux*, 1842. Guéniot stated that these knots are rarely drawn sufficiently tight to interfere with the circulation and produce dangerous effects upon the fœtus. This results from the material with which the cord is smeared, and which prevents its constriction so perfectly that after delivery, if it be knotted artificially, it will be found difficult to tie it tightly unless this smegma be first removed. Besides, the incessant motion of the arterial fluid tends to prevent the accident by loosening the knot. In this case death was probably determined by the three turns about the neck which increased the vascular disturbance.

2. *Tubal Pregnancy.* Albu. (*Berlin Klin. Wochenschr.*, Feb., 1876.)

A woman was seized with vertigo, malaise, diarrhœa, and violent hypogastric pain, followed by repeated syncope. Up to date her health had been perfect. She had not menstruated for two months. The abdomen and the uterus were exquisitely sensitive to the touch. As she was living in concubinage, poisoning was thought to be the cause. Shortly after the visit of her physician, she went into collapse and died.

The autopsy revealed rupture of the left fallopian tube, from which an hæmorrhage had occurred. The membrane of the ovum was also torn, the latter having been fecundated and increased to the size of a nut.

Albu remarked that Maschka had already called attention to the symptoms of poisoning exhibited in cases of pregnancy of the tube, viz: abdominal pain, extreme feebleness, nausea, pallor of the surface, smallness of the pulse, collapse, cramps, convulsions and sudden death.

Heim, in his thesis on pregnancies of the fallopian tube, gives the details of 32 cases observed by himself.

3. *Uterine Mucous Membrane Expelled without Metrorrhagia—Probable Extra-uterine Pregnancy.* HUTINEL. (*Le Progrès Méd.*, No. 7.)

A woman, 25 years of age, had severe pelvi-peritonitis after her second confinement. She was well for two months when she had menstrual suppression. She then experienced some pain, her limbs became heavy. She grew larger, and had dyspepsia without vomiting. These troubles were temporarily relieved when she had violent abdominal pain, syncope, bilious vomiting, abdominal distension, and an exsanguine facies without apparent loss of blood. Acute generalized peritonitis followed.

On July 26, her skin was of a waxy yellow hue, and the conjunctivæ, gums and lips were almost bloodless. The belly was equally distended in all directions and so sensitive that she could not endure the contact of the hand. Some greenish vomiting; pulse, 120; temp. 40° C.

On July 28, there was relief of symptoms and a triangular membrane was removed from the vagina, half a centimetre thick, villous upon each face, slightly mamelonnated, but smooth, and presenting pitted depressions which could be recognized as the glandular orifices of a section of uterine mucous membrane—a recognition confirmed by the microscope.

The distinct symptoms of internal hæmorrhage preceding the peritonitis and the expulsion of the membrane without hæmorrhage externally, pointed to extra-uterine pregnancy.

4. *Hydatids of the Womb.* McCLELLAND. (*Rich. & Lou. Med. Jour.*, Feb.)

In the *Richmond and Louisville Med. Journal*, Feb., Dr. M. A. McClelland has reported an instance of this rare disease. The patient, a primipara, aged 34 years, thought herself five months advanced in pregnancy. Had been flowing for several days when first seen; paroxysmal pains; os somewhat opened, through which an uneven mass was felt. The expulsion of the fœtus was soon followed by that of the diseased placenta which was made up of cysts filled with clear serum and varying in size from that of a mustard seed to that of a Concord grape. The fœtus was well formed, some ten inches in length, and gasped a number of times.

5. *Rupture of Uterus.* FULLER. (*Cin. Lan. & Obs.*, March.)

Dr. S. W. Fuller reports this case in *Cin. Lancet & Obs.*, March. The patient was a stout woman with pendulous belly, who was already the mother of five children. When first seen the os was well dilated and the membranes ruptured. Six hours subsequently the head was just entering the strait in first position of vertex, when suddenly the pains ceased, the patient complained of pain in the region of the fundus uteri, and grew very restless. An attempt to apply forceps failed, and turning was decided upon. The lower extremities of the fœtus were found quite in the abdominal cavity, but version and delivery were not difficult. The hand was subsequently passed through a very extensive rupture of the uterus into the peritoneal cavity; no clots were found; the placenta was lying loose within the uterus; the organs were contracted. The injury was followed by high fever, but at the end of seven weeks the patient was able to sit up.

Rather more than two years after this accident which has been described, the patient safely gave birth to a six-months fœtus; the placenta being everywhere firmly adherent.

6. *Presentation of Four Hands.* STIEBELING. (*N. Y. Med. Journal.*)

Dr. Stiebeling was called to the case; the membranes were intact; os well dilated. The presentation of hands could be distinctly made out, and it was decided to perform version; upon rupturing the membranes, two hands were met, and in going higher two other hands were encountered, and then two heads. A foot was pulled down and the delivery of a boy soon followed; the second fœtus, also a male, was soon delivered in the same manner. Mother and children did well.

7. *Hæmorrhage from Abortion.* PALLEN. (*N. Y. Med. Rec.*, Feb. 12.)

Concerning hæmorrhage from abortion, Dr. Pallen said, before the New York Academy of Medicine, (*N. Y. Med. Rec.*, Feb. 12), that he always dilated the cervix with his hand or other means, and removed the fœtus and placenta at once, as a guard against hæmorrhage and septic infection.

In the discussion of Dr. Pallen's remarks, Dr. Mundé said that he was of the opinion that no harm would follow if the placenta was left in the uterus for a number of days; it would, as a rule, soon be found in the vagina.

Dr. Hubbard said that for twenty years he had left the placenta of abortion entirely alone. If hæmorrhage is severe, he uses a tampon. In the great majority of cases, the placenta will be found loose in the vagina at the end of the second day. He regards this much safer practice than forcibly opening the cervix uteri for the removal of the placenta.

8. *Induced Lactation.* GILBERT. (*Louisv. Med. News*, March 11.)

An instance of this is reported by Dr. Gilbert, (*Louisville Medical News*, March 11). The subject, a married and childless woman, took an orphan child three weeks old. Artificial feeding disagreeing with the infant, the woman, whose desire for children was strong, was induced to allow the infant to take her nipple. A poultice of leaves from the castor oil plant was applied to the breasts, and teaspoonful-doses of castor oil were given every three hours. At the end of twenty-four hours, a peculiar sensation was experienced in the breasts, and in three days the function of lactation was well established. The infant thrived well.

9. *Medico-legal Evidence of Independent Life in a New Born Child.*

In a little pamphlet with this title, Dr. Gaston (Montgomery, Ala.) has called attention to the fact that the function of respiration alone is evidence of extra-uterine life; and that the fact that the fœtal heart has pulsated after birth is not sufficient evidence in a medico-legal point of view.

10. *Gestation Extending Three Hundred and Six Days.*

Dr. Graves reports the case in *Boston Med. & Surg. Journal*, March 30 The subject, seventeen years old, was the wife of a sea-faring man. Three days before the husband sailed, the woman ceased to menstruate; sexual intercourse was had each of the two nights following. Three weeks after her husband left her, she had nausea, and Dr. Graves thought her pregnant. At the expiration of nine months, the Doctor was asked to hold himself in readiness to attend upon the woman as she was having some pain. During the night of the three hundred and sixth day from the departure of her husband the labor really began, and continued four hours; the woman giving birth to a healthy male child weighing ten and one-half pounds.

11 *The Physiological Cause of Muscular Contraction of the Gravid Uterus.*

Dr. Thomas Mays, (*N. Y. Med. Jour.*, Feb.,) in an interesting paper on this subject advances the idea that it is a condition of bloodlessness of the uterus during the last days of pregnancy which induces the contractions of the organ during that period, these contractions growing more forcible until labor has begun. He finds the explanation of this deficient blood supply to the uterus in the encroachment which the heavy gravid uterus makes upon the uterine supply vessels, by which the circulation through them is impeded. It is a little significant that this encroachment is greatest as term is approached, and coincident with the beginning of uterine contractions, when the lower part of the body of the uterus begins to contribute most to the development of the uterine cavity.

